# Effect of single tablet of fixed-dose amlodipine and atorvastatin on blood pressure/lipid control, oxidative stress, and medication adherence in type 2 diabetic patients

**DOI:** 10.1186/1758-5996-6-56

**Published:** 2014-05-18

**Authors:** Masami Tanaka, Risa Nishimura, Takeshi Nishimura, Toshihide Kawai, Shu Meguro, Junichiro Irie, Yoshifumi Saisho, Hiroshi Itoh

**Affiliations:** 1 Department of Internal Medicine, School of Medicine, Keio University, Tokyo, Japan

**Keywords:** Malondialdehyde-modified low-density lipoprotein, Oxidative stress, Amlodipine, Atorvastatin, Diabetes mellitus, Hypertension, Dyslipidemia, Atherosclerosis, Medication adherence, Combination tablet

## Abstract

**Background:**

Oxidized low-density lipoprotein (LDL) plays central roles in the formation and progression of atherosclerotic lesions. Malondialdehyde (MDA)-modified LDL (MDA-LDL) is speculated to be generated as a result of oxidative stress in the human body. Because both amlodipine and atorvastatin have been reported to reduce oxidative stress, it is expected that both drugs would have a favorable influence to reduce oxidative stress.

**Objective:**

The objective of this study was to investigate the effects of a single pill of amlodipine (5 mg)/atorvastatin (10 mg) on oxidative stress, blood pressure/lipid control and adherence to medication in patients with type 2 diabetes.

**Methods:**

This combination tablet was administered to 29 patients (16 male), and MDA-LDL, blood pressure, lipid profile, renal/liver function, CPK, hs-CRP, adiponectin, BNP, and HbA1c were measured at baseline, 6, and 12 months, and baPWV and mean IMT were measured at baseline and 12 months. Medication adherence was examined using a questionnaire at 6 months.

**Results:**

MDA-LDL was decreased significantly. LDL-C, TG, and Cr were significantly decreased at 6 and 12 months compared with baseline. eGFR was increased at 6 months, and urinary albumin/creatinine ratio was decreased at 12 months. BNP was decreased at 6 and 12 months, and adiponectin was increased at 12 months. Both mean IMT and baPWV were significantly decreased. The results of the questionnaire showed that 93% of patients were satisfied with this medication. No severe adverse event was observed.

**Conclusion:**

This combination tablet controlled both hypertension and dyslipidemia well in type 2 diabetic patients. The deceases in mean IMT and baPWV might suggest the improvement of atherosclerosis by this medication, which could be caused by the reduction of oxidative stress measured by MDA-LDL. In addition, this medication is expected to improve medication adherence.

## Background

Oxidized low-density lipoprotein (LDL), which is produced through modification of LDL by oxidation, plays central roles in the formation and progression of atherosclerotic lesions [1]. Malondialdehyde (MDA)-modified LDL (MDA-LDL), a representative oxidized LDL [2], is speculated to be generated as a result of oxidative stress in the human body. It is suggested that MDA-LDL is involved in many processes of atherosclerosis such as vascular intima injury and foam cell formation [3,4]. Therefore, MDA-LDL has attracted attention as a risk factor for macrovascular disease, such as ischemic heart disease and cerebral infarction.

Investigation of diabetic patients with a history of coronary heart disease has shown that those with increased plasma MDA-LDL concentration have a significantly higher risk of coronary heart disease [5]. Moreover, it has been shown that the risk of restenosis after percutaneous coronary intervention is high if the concentration of MDA-LDL is elevated [6]. It is reported that MDA-LDL concentration in patients with cerebral infarction is significantly higher than that in those without, at any age [7]. In addition, type 2 diabetes mellitus per se is an important risk factor for macrovascular disease irrespective of the history of coronary heart disease or cerebrovascular disease [8]. Therefore, it is suggested that measurement of the serum concentration of MDA-LDL would be useful for the prevention of coronary heart disease and cerebrovascular disease in patients with type 2 diabetes mellitus.

Both amlodipine, which is used to treat hypertension and angina pectoris, and atorvastatin, which is used to treat hypercholesterolemia/familial hypercholesterolemia, have been reported to reduce oxidative stress [9]. Therefore, it is expected that both drugs would have a favorable influence to reduce oxidative stress. Moreover, there is accumulating evidence that these medications can be used for the prevention of coronary heart disease and cerebral infarction [10-13].

In this study, we prospectively investigate the impact of combination therapy with amlodipine and atorvastatin on plasma MDA-LDL level in patients with type 2 diabetes mellitus and concomitant hypertension and hypercholesterolemia. We used a fixed combination tablet containing 5 mg amlodipine and 10 mg atorvastatin. This study hypothesizes that simplification of patients’ prescribed medication by using a combination tablet will improve medication adherence. Therefore, in this study, a questionnaire survey about medication adherence was also conducted.

### Subjects and methods

#### Subjects

The subjects were 29 Japanese outpatients with type 2 diabetes (16 men and 13 women) with complications of hypertension and hypercholesterolemia aged 20 years or older. Patients who could not achieve the lipid control goal of the Japan Atherosclerosis Society (JAS) 2012 Guidelines for prevention of atherosclerotic cardiovascular disease (LDL-cholesterol (LDL-C) < 100 mg/dl for patients with prior coronary heart disease and LDL-C < 120 mg/dl for patients without prior coronary heart disease) [14] and/or who could not achieve the blood pressure-lowering goal of the 2009 Japanese Society of Hypertension guidelines for the management of hypertension (<130/80 mmHg) [15] in spite of taking a statin and/or calcium channel blocker for at least three months were enrolled.

## Methods

MDA-LDL, blood pressure, LDL-C, high-density lipoprotein cholesterol (HDL-C), triglyceride (TG), serum creatinine (Cr), estimated glomerular filtration ratio (eGFR), urinary albumin-creatinine ratio (ACR), aspartate aminotransferase (AST), alanine aminotransferase (ALT), γ-glutamyl transferase (GTP), creatine phosphokinase (CPK), high-sensitivity C-reactive protein (hs-CRP), adiponectin, brain natriuretic peptide (BNP) and glycosylated hemoglobin A1c (HbA1c) were measured at baseline and 6 and 12 months after the start of administration of this combination tablet and compared. MDA-LDL was measured using an enzyme-linked immunosorbent assay as reported previously [6,16]. LDL-C was calculated using the Friedewald formula (LDL-C = total cholesterol (TC)-HDL-C-TG/5). ACR was measured by a turbidimetric immunoassay with a Superior-Microalbumin kit (Daiichi Pharmaceutical Co., Tokyo, Japan) and the Jaffé reaction, using an automated analyzer. hs-CRP was measured by a latex particle-enhanced immunoassay with the nephelometry method (LABOSPECT 008; Hitachi, Tokyo, Japan), using an hs-CRP kit from Mitsubishi Chemical Medicine (Tokyo, Japan). BNP was measured by a fluorescence enzyme immunoassay, using a monoclonal antibody (E-test, Tosoh II BNP; Tosoh, Tokyo, Japan). Adiponectin was measured by a latex particle-enhanced immunoassay as reported previously [17]. HbA1c was determined by HPLC (Toso, Tokyo, Japan) and presented as the equivalent National Glycohemoglobin Standardization Program (NGSP) value.

Brachial-ankle pulse wave velocity (baPWV) and mean intima-media thickness (IMT) were measured at baseline and 12 months after the start of administration and compared. baPWV was measured after 5 min of bed rest using a BP-203RPE III (form PWV/ABI) device (Omron Healthcare, Kyoto, Japan). IMT was measured by ultrasonography B-mode imaging, using a PowerVision® 6000 ultrasound machine (Toshiba, Tokyo, Japan).

During the study period, the type and dose of medications that might influence glycemic, blood pressure and/or lipid control were not changed in principle. We verified eligibility of the patients at outpatient medical examinations by asking them whether they took this combination tablet as instructed by the doctors in charge.

In order to investigate medication adherence to this combination tablet, we conducted a questionnaire survey. The questionnaire consisted of questions about 1) the degree of satisfaction with taking the combination tablet, 2) reason(s) why the patient thinks it is good to take the combination tablet, 3) desire to continue taking the combination tablet in the future, and 4) feelings about the reduction in the number of tablets the patient is taking. They wrote the answers to the multiple choice questions themselves.

In order to investigate the effects of patient characteristics on medical adherence, we performed multivariate analyses. We scored the results of the questionnaire as follows: ‘Do you think it is good to take this combination tablet?’: very good 3, good 2, not good 1; ‘Do you want to continue taking this combination tablet?’: yes, very much 4, I think so 3, not very much 2, absolutely not 1; ‘Do you think that even a decrease in one medication is a good thing?’: yes, very much 4, I think so 3, not very much 2, I completely disagree 1. Then, stepwise regression analyses were performed using the following factors as independent variables: gender (men = 1, women = 2), age, duration of diabetes, BMI, and baseline HbA1c.

This study was approved by the ethical committee of Keio University School of Medicine. Doctors gave the patients a sufficient explanation of this clinical study. After confirming that the patients understood and agreed to take part, written informed consent was obtained.

### Statistical analysis

Results were expressed as mean ± standard deviation. Data analyses were performed using the JMP® software package, version 6 (SAS Japan Institute Ltd., Tokyo, Japan) for Windows®. Paired *t*-test was used to assess the significance of differences between values obtained before and 6 and 12 months after the start of administration of the combination tablet. Values of p < 0.05 were considered statistically significant (two-tailed analysis).

## Results

Baseline characteristics of the patients are shown in Table 1. Age, body mass index, duration of diabetes and HbA1c were 58.9±11.0 years, 26.2±5.4 kg/m^2^, 14.4±6.2 years, and 8.3±1.6%, respectively. Many patients had complications of micro- and macrovascular disease.

**Table 1:** Patient characteristics at baseline (n = 29)

**Gender (n)**	**Male 16 Female 13**	**Hypoglycemic agent (n)**	
**Age (years)**	**58.9±11.0**	**OHA**	**16**
**BMI (kg/m**^**2**^**)**	**26.2±5.4**	**Insulin**	**2**
**Diabetes duration (years)**	**14.4±6.2**	**OHA+insulin**	**10**
**HbA1c (%)**	**8.3±1.6**	**Antihypertensive agent (n)**	
**Diabetic complications (n)**		**CCB**	**17**
** Neuropathy**	**18**	**ARB**	**21**
** Retinopathy**	**9**	**ACE inhibitor**	**4**
** Nephropathy**	**13**	**Diuretic**	**3**
**Macrovascular disease (n)**		**Β blocker**	**2**
** Cerebral infarction**	**4**	**Antihyperlipidemic agent (n)**	
** Angina/myocardial infarction**	**4**	**Statin**	**17**
		**Ezetimibe**	**1**
		**Antiplatelet agent (n)**	
		**Aspirin**	**5**
		**Clopidogrel**	**1**

The results are shown as mean ± standard deviation, or number of patients.

Each patient belongs to only one group of diabetic agent.

OHA: oral hypoglycemic agent.

**Figure 1:**
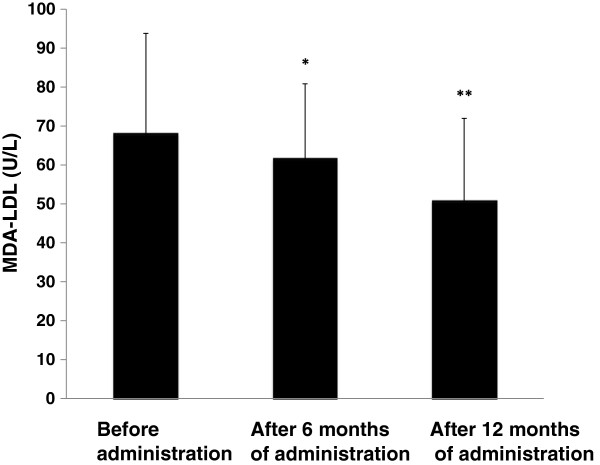
**Change in plasma MDA-LDL concentration.** The results are shown as mean±standard deviation. Comparison with the value before administration of the combination tablet. *p < 0.05, **p < 0.01 (paired *t*-test).

As shown in Figure 1, after administration of the combination tablet, MDA-LDL decreased significantly compared with baseline (69.2±25.7 at baseline→59.3±18.3 at 6 months (p < 0.05)→51.4±21.4 U/L at 12 months (p < 0.01)). Both systolic and diastolic blood pressure decreased significantly (systolic: 150.2±12.2 at baseline→136.2±17.4 at 6 months (p < 0.01)→131.5±10.2 mmHg at 12 months (p < 0.01), diastolic: 87.3±9.2 at baseline→82.0±9.3 at 6 months (p < 0.01)→78.0±6.6 mmHg at 12 months (p < 0.01)). LDL-C decreased significantly from 110.8±33.7 mg/dl at baseline to 82.3±26.7 mg/dl at 6 months (p < 0.01), and to 81.3±25.1 mg/dl at 12 months (p < 0.01). HDL-C did not change significantly (48.9±12.7 mg/dl at baseline→49.3±13.6 mg/dl at 6 months→47.7±13.9 mg/dl at 12 months). On the other hand, TG decreased significantly (193.9±127.9 mg/dl at baseline→144.7±71.0 mg/dl at 6 months (p < 0.05)→151.2±91.6 mg/dl at 12 months (p < 0.01)).

Compared with baseline, serum Cr was decreased significantly at both 6 and 12 months (0.77±0.19 mg/dl at baseline→0.73±0.17 mg/dl at 6 months (p < 0.01)→0.74±0.19 mg/dl at 12 months (p < 0.05)), and eGFR was increased significantly at 6 months (77.0±30.4 at baseline→80.2±27.4 at 6 months (p < 0.01)→78.6±25.0 ml/min/1.73 m^2^ at 12 months). ACR was decreased significantly at 12 months (67.6±118.4 at baseline→42.9±70.5 at 6 months→39.5±67.2 mg/gCr at 12 months (p < 0.05)).

As shown in Table 2, AST, ALT, γGTP, CPK, hs-CRP, and HbA1c did not change significantly during the observation period.

**Table 2:** Changes in clinical parameters

	**Before administration**	**After 6 months of administration**	**After 12 months of administration**
**AST (IU/L)**	**27.8±13.5**	**26.5±12.7**	**24.8±11.1**
**ALT (IU/L)**	**31.8±23.6**	**30.8±18.2**	**26.5±14.0**
**γ-GTP (IU/L)**	**44.7±42.0**	**48.0±49.6**	**45.7±51.8**
**CPK (IU/L)**	**109.6±57.3**	**109.8±53.0**	**109.2±69.3**
**hs-CRP (mg/dL)**	**0.13±0.18**	**0.11±0.15**	**0.10±0.13**
**HbA1c (%)**	**8.3±1.6**	**8.5±1.5**	**8.3±1.6**

The results are shown as mean **±** standard deviation.

No parameter showed a significant change during the course of this study.

**Figure 2:**
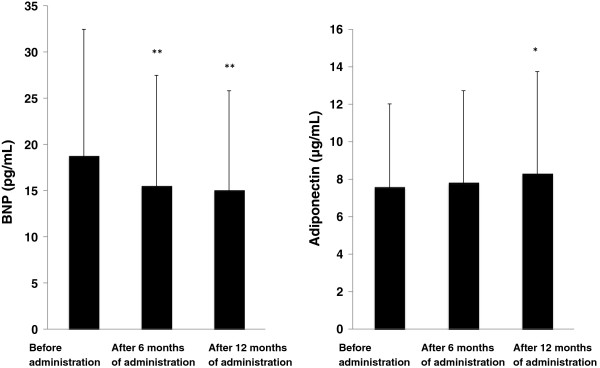
**Changes in plasma concentration of BNP and adiponectin.** The results are shown as mean ± standard deviation. Comparison with the value before administration of the combination tablet. *p < 0.05, **p < 0.01 (paired *t*-test).

As shown in Figure 2, BNP was significantly decreased at both 6 and 12 months (19.5±14.0 at baseline→15.4±12.5 at 6 months (p < 0.01)→15.3±11.2 pg/ml at 12 months (p < 0.01)), and adiponectin was increased at 12 months (7.64±4.49 at baseline→7.82±4.73 at 6 months→8.15±5.25 μg/ml at 12 months (p < 0.05)).

**Figure 3:**
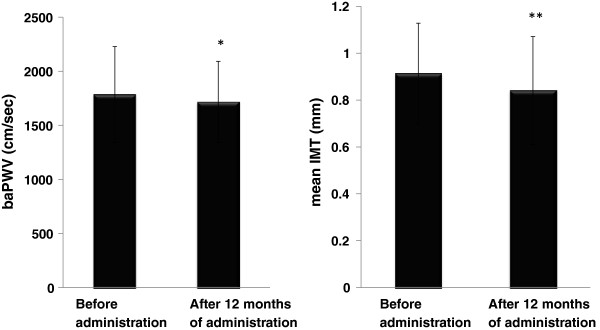
**Changes in baPWV and mean IMT.** The results are shown as mean ± standard deviation. Comparison with the value before administration of the combination tablet. *p < 0.05, **p < 0.01 (paired *t*-test).

As shown in Figure 3, compared with baseline, both baPWV and mean IMT improved significantly (baPWV: 1768.0±477.4 at baseline→1689.9±389.5 cm/sec at 12 months (p < 0.05), mean IMT: 0.91±0.21 at baseline→0.82±0.24 mm at 12 months (p < 0.01)).

The results of the questionnaire survey are shown in Table 3. Answers were obtained from 27 patients. Of the patients, 93% (25/27) were satisfied with this combination tablet (3a). The most common reason for satisfaction was that “LDL-C was decreased sufficiently”. The second most common reason was that “blood pressure was decreased sufficiently” (3b).

**Table 3:** Results of questionnaire survey on medication adherence

**a. Do you think it is good to take this combination tablet?**	
**Very good**	**8**
**Good**	**17**
**Not good**	**0**
**No answer**	**2**
**b. Reason(s) why you think that it was good to take this combination tablet**	
**LDL-C was decreased sufficiently**	**12**
**Blood pressure was decreased sufficiently**	**10**
**Tablet is small and easy to swallow**	**8**
**I do not have to discriminate morning and evening doses**	**5**
**Number of times taking medication is reduced**	**5**
**Number and dose of medications taken in one day is reduced**	**6**
**Number and dose of medications taken at one time is reduced**	**8**
**c. Do you want to continue taking this combination tablet?**	
**Yes, very much**	**12**
**I think so**	**14**
**Not very much**	**0**
**Absolutely not**	**0**
**No answer**	**1**
**d. Do you think that even a decrease in one medication is a good thing?**	
**Yes, very much**	**19**
**I think so**	**5**
**Not very much**	**1**
**I completely disagree**	**0**
**I have no idea**	**2**

N: number of answers.

3b: Multiple answers were allowed.

Almost all (26/27) patients answered that they wanted to continue taking this medication (3c), and 89% (24/27) answered that even a decrease of one tablet is a good thing (3d).

Stepwise regression analyses demonstrated that none of the factors investigated was identified as an independent determinant of the scores of three questions (1): gender β = 0.15, p = 0.54; age β = -0.20, p = 0.58, duration of diabetes β = 0.04, p = 0.90, BMI β = -0.25, p = 0.30, baseline HbA1c β = 0.22, p = 0.45, 3): gender β = 0.12, p = 0.60, age β = 0.47, p = 0.14, duration of diabetes β = -0.32, p = 0.30, BMI β = 0.068, p = 0.78, baseline HbA1c β = 0.45, p = 0.13; 4): gender β = 0.24, p = 0.33, age β = -0.61, p = 0.086, duration of diabetes β = 0.35, p = 0.31, BMI β = -0.042, p = 0.87, baseline HbA1c β = -0.29, p = 0.35).

No severe adverse event occurred, and all 29 patients could continue taking this combination tablet.

## Discussion

After the administration of a fixed combination tablet containing 5 mg amlodipine and 10 mg atorvastatin, blood pressure, LDL-C, TG, and MDA-LDL were significantly improved in type 2 diabetic patients with hypertension and dyslipidemia. In addition, baPWV and mean IMT, which are markers of atherosclerosis, also improved.

The achieved LDL-C, systolic and diastolic blood pressure were 81.3±25.1 mg/dl,131.5±10.2 mmHg, and 78.0±6.6 mmHg, respectively. LDL-C (<120 mg/dl) and diastolic blood pressure (<80 mmHg) reached the target in the Japanese guidelines, and systolic blood pressure almost reached the goal (<130 mmHg). Therefore, it was shown that this combination tablet could control both hypertension and hypercholesterolemia, which are the two major risk factors for atherosclerosis. Taken together with the report that thickening of IMT is an independent, significant predictor of cerebral infarction and coronary artery disease [18,19], this medication is considered to be very useful for the prevention of macrovascular complications in type 2 diabetic patients.

In the Anglo-Scandinavian Cardiac Outcomes Trial-Lipid Lowering Arm (ASCOT-LLA), it was shown that treating hypercholesterolemia could prevent coronary heart disease in hypertensive patients taking antihypertensive medication “at high risk”, even though their total cholesterol was 250 mg/dl or less [13]. Patients “at high risk” means that they have several characteristics including old age (55 years or more), male, smoking habit, type 2 diabetes, microalbuminuria, and left ventricular hypertrophy. Therefore, the results of ASCOT-LLA can be applied to many patients we encounter in daily clinical settings. This indicates the usefulness of treating not only hypertension but also dyslipidemia at the same time in hypertensive patients.

Another analysis of ASCOT-LLA showed that the incidence of cardiovascular events was significantly decreased in patients treated with amlodipine and atorvastatin compared with patients treated with atenolol and atorvastatin, even though there was no significant difference in blood pressure, TC, or LDL-C between the two patient groups [20]. Consequently, from the perspective of cardiovascular event prevention, it was suggested that the combination of amlodipine and atorvastatin might have high efficacy and that this fixed-dose combination tablet is very important because patients can take both amlodipine and atorvastatin by taking only one tablet.

Because MDA-LDL is thought to be involved in many steps of atherosclerosis, it is a promising candidate as a risk factor for atherosclerosis other than established risk factors such as hypertension, diabetes, and LDL-C. It has been shown that the plasma concentration of MDA-LDL is increased in patients with unstable carotid artery plaques [21]. In the present study, this combination tablet decreased MDA-LDL, which suggests the possibility that this medication might directly intervene in many processes of atherosclerosis. Taking these findings together with the result that this combination tablet improved baPWV and mean IMT, this medication is thought to be highly promising as an anti-atherosclerosis drug.

MDA-LDL has been reported to exert direct cytotoxicity on endothelial cells, to promote synthesis and secretion of adhesion molecules, to increase platelet aggregation and monocyte adhesion, and to enhance foam cell formation in atherosclerotic lesions, all of which lead to remodeling of vessel walls [3,4]. The level of circulating MDA-LDL is reported to be elevated in patients with diabetes [22]. The mechanisms underlying the increase in MDA-LDL in diabetic patients are not clear, although enhancement of lipid peroxidation might be involved [23]. The main causes of death of diabetic patients are macrovascular diseases caused by severe atherosclerosis. Considering this, the observation that this combination tablet decreased MDA-LDL significantly might have clinical significance from the perspective of macrovascular disease prevention. Measurement of MDA-LDL, especially in diabetic patients, is expected to have a clinical impact.

During the observation period, Cr and ACR decreased, eGFR increased, and BNP decreased significantly. All of these results may reflect the reno- and cardio- protective effects of this combination tablet. Although statins have been reported to possess a reno-protective effect, it was shown that the reno-protective effect of atorvastatin is stronger than that of other statins [24]. Atorvastatin has been shown to improve eGFR and Cr clearance in several sub-analyses from large scale clinical trials such as the Collaborative Atorvastatin Diabetes Study (CARDS) [25], the Treating to New Targets (TNT) [26], and the Greek atorvastatin and coronary heart disease evaluation (GREACE) [27].

As one of the mechanisms underlying the reno-protective effect of atorvastatin, the possibility that atorvastatin might suppress podocyte injury was reported recently [28]. In a sub-analysis of TNT, administration of atorvastatin significantly reduced hospitalization due to heart failure [29]. Therefore, as a mechanism of BNP lowering in this study, there is a possibility that the pleiotropic effect of atorvastatin was added to the blood pressure-lowering effect of amlodipine.

In the present study, it was shown that the concentration of adiponectin was increased after 12 months of administration of this combination tablet. It is reported that when both atorvastatin and amlodipine were administered in patients with coronary heart disease, hypertension and dyslipidemia, the plasma concentration of adiponectin increased [30]. In this study, it is also reported that the increase in adiponectin concentration and improvement ratio of vascular intima function evaluated by flow-mediated vasodilation (FMD) method were correlated [30]. Although the underlying mechanism of the improvement of baPWV and mean IMT in this study is not clear, increase in adiponectin might be involved, through an anti-atherosclerotic action, in addition to blood pressure lowering by amlodipine and LDL-C lowering by atorvastatin.

An improvement in medication adherence leads to an increase in the direct benefit of the medication. It has been reported that as the number of drugs taken in one day increases, medication adherence deteriorates, and that as the number of drugs and times of medication decrease, medication adherence improves [31]. Moreover, it is reported that medication adherence is improved by using a combination tablet [32].

The questionnaire survey about medication adherence in the present study showed high satisfaction with the combination tablet. As a reason for this, many patients stated a sufficient decrease in LDL-C and blood pressure. It is important that patients evaluated both the decrease in number of tablets and the drug efficacy, that is, improvement in blood pressure and dyslipidemia highly. Because 89% (24/27) of patients answered that even a decrease in one tablet is a good thing, the medication adherence to this combination tablet might be very good. Stepwise regression analyses showed that none of gender, age, duration of diabetes, BMI, and baseline HbA1c influenced the results of the questionnaire survey. This might suggest the possibility that this medication improves medication adherence in many patients with a wide variety of backgrounds. However, this should be confirmed by larger scale studies containing a control group. It is reported that if medication adherence is high for 1–2 years after initiation, medication adherence will continue to be high thereafter [33]. Therefore, it is expected that the participants in this clinical trial will continue to take this combination tablet in the future.

This study included many patients with a long duration of diabetes mellitus or poor glycemic control. Many patients had complications of obesity, diabetic microangiopathy, and macroangiopathy. Many patients took an antihypertensive, antidiabetic, or antiplatelet drug. Notwithstanding, no severe adverse event occurred, and all 29 patients could continue taking this combination tablet. Liver function, CPK, and HbA1c did not deteriorate during the course of the study. Therefore, the safety of this medication appears to be confirmed in high risk patients.

There are some limitations of this study. Firstly, the number of patients included was small. Secondly, this was a single arm study and did not have a control. Finally, the results of our study may not be applicable to the general population or to patients with type 2 diabetes in a primary care setting, because patients who attend university hospitals are selected patients.

## Conclusion

It was shown that a single-pill of amlodipine (5 mg)/atorvastatin (10 mg) could control both blood pressure and lipid profile well in type 2 diabetic patients with complications of hypertension and dyslipidemia. The deceases in mean IMT and baPWV might suggest the improvement of atherosclerosis by this medication, which could be explained at least in part by the reduction of oxidative stress measured by MDA-LDL. This medication is expected to prevent cardiovascular events through a decrease of oxidative stress and improvement of medication adherence.

## Competing interests

The authors declare that they have no competing interests.

## Authors' contribution

MT, RN and TN disigned and performed this clinical study. TK wrote the paper. SM performed the statistical analyses. JI and YS recruited the patients. HI surpervised the project. All authors read and approved the final manuscript.
